# Managing Iatrogenic Common Femoral Artery Hemorrhage in an Extracorporeal Membrane Oxygenation (ECMO) Patient When Standard Access Fails

**DOI:** 10.7759/cureus.107898

**Published:** 2026-04-28

**Authors:** Renato Abu Hana, Ruben G Ortiz Cordero, Oswaldo A Guevara Tirado, Vedant Garg, Vinicius Adami Vayego Fornazari

**Affiliations:** 1 Radiology, University of Florida College of Medicine, Jacksonville, USA; 2 Radiology, University of Florida College of Medicine, Gainesville, USA

**Keywords:** endovascular repair, iatrogenic vascular injury, retrograde pedal access, transradial access, venoarterial extracorporeal membrane oxygenation (va ecmo)

## Abstract

Iatrogenic injuries related to resuscitative efforts and emergent vascular access can be life-threatening, particularly in critically ill patients with limited treatment options. We present a case of a patient who collapsed due to an acute ST-elevation myocardial infarction and underwent prolonged resuscitation, which was complicated by hepatic injury likely secondary to chest compressions and injury to the left common femoral artery (CFA) following multiple arterial line attempts.

The patient was referred for an angiogram evaluation primarily for suspected hepatic injury. Conventional femoral access was not feasible, as the right groin was occupied by emergent venoarterial extracorporeal membrane oxygenation (VA-ECMO) cannulation, and a femoral compression device had been applied to the left groin following failed arterial access. Therefore, hepatic angiography was performed via left transradial access and demonstrated no active hemorrhage. Given the history of difficult arterial femoral access, further interrogation of the left iliofemoral system was undertaken, revealing multiple sites of active bleeding from the left CFA. Given the patient’s clinical instability and poor candidacy for surgical repair, an endovascular approach was pursued. However, the available covered stent system could not be advanced to the target from the radial access due to length limitations, necessitating an alternative strategy. Retrograde pedal access was obtained, allowing successful treatment of the injured CFA segment with restoration of hemostasis and preservation of distal perfusion. The patient experienced no recurrent bleeding, limb ischemia, or need for reintervention during the hospitalization. This case highlighted the spectrum of resuscitation-related iatrogenic injuries and underscores the importance of adaptable endovascular strategies when conventional access routes are not feasible.

## Introduction

Iatrogenic injuries of the common femoral artery (CFA) are potentially serious complications of percutaneous vascular access, particularly during emergent resuscitation efforts [1,2]. When such injuries occur, hemorrhage control can be challenging. Surgical repair carries a high risk in critically ill patients, and manual compression may be insufficient or may exacerbate bleeding at the site of injury [3]. These constraints can significantly limit available treatment options and necessitate alternative strategies for hemorrhage control. Endovascular repair with covered stent-grafts has emerged as a less invasive alternative to open surgical management; however, its success depends on the availability of suitable access routes for device delivery [1,4,5].

Transfemoral access remains the conventional route for iliofemoral interventions but may be precluded by the presence of indwelling devices, such as extracorporeal membrane oxygenation (ECMO) cannulas, or by compression devices applied at the site of injury [6,7]. In such cases, transradial access (TRA) can serve as an effective alternative for diagnostic angiography [7,8]. However, TRA is often limited in therapeutic interventions due to the shaft length of devices required to reach lower-extremity targets [7,8].

Despite the increased use of transradial access as an alternative in complex endovascular procedures, current literature provides limited guidance for the specific scenario in which transradial access is available but insufficient for covered stent delivery due to catheter shaft length constraints and femoral vascular access is unavailable [7,9]. In this report, we present a case of iatrogenic left common femoral artery (CFA) hemorrhage following attempted arterial line placement during resuscitation for an acute ST-elevation myocardial infarction. Vascular access options were severely limited due to competing constraints in both groins, precluding conventional approaches. This case highlighted a stepwise endovascular strategy for managing complex vascular injury when standard access routes are not feasible and aimed to describe a salvage retrograde pedal access approach as a viable alternative when both conventional transfemoral and transradial routes are not viable.

## Case presentation

A 50-year-old man with a history of diabetes mellitus presented after a witnessed collapse associated with acute dyspnea. Prehospital evaluation revealed hypoxia and an inferoseptal ST-elevation myocardial infarction. He developed ventricular fibrillation and cardiac arrest en route, requiring multiple defibrillations and advanced cardiac life support with return of spontaneous circulation.

On arrival, vital signs were remarkable for hypertension (155/103 mmHg), tachycardia (139 beats per min), tachypnea (22 breaths per min), and hypoxemia (peripheral oxygen saturation of 53%). Due to refractory cardiogenic shock, the patient was emergently cannulated for venoarterial extracorporeal membrane oxygenation (VA-ECMO) via the right common femoral artery and vein. During resuscitation, multiple unsuccessful attempts were made to obtain arterial access in the left groin, and no arterial sheath was successfully placed. Due to bleeding from the attempted access site, a femoral compression device (FemoStop Gold; Temecula, CA: Abbott) was placed over the left groin for hemostasis. The presence of the compression device limited local physical examination of the left groin and complicated the assessment of hematoma formation or signs of vascular injury. Furthermore, during resuscitative efforts, the patient became hypotensive (50/35 mmHg) and required vasopressor support with an epinephrine drip.

**Figure 1:**
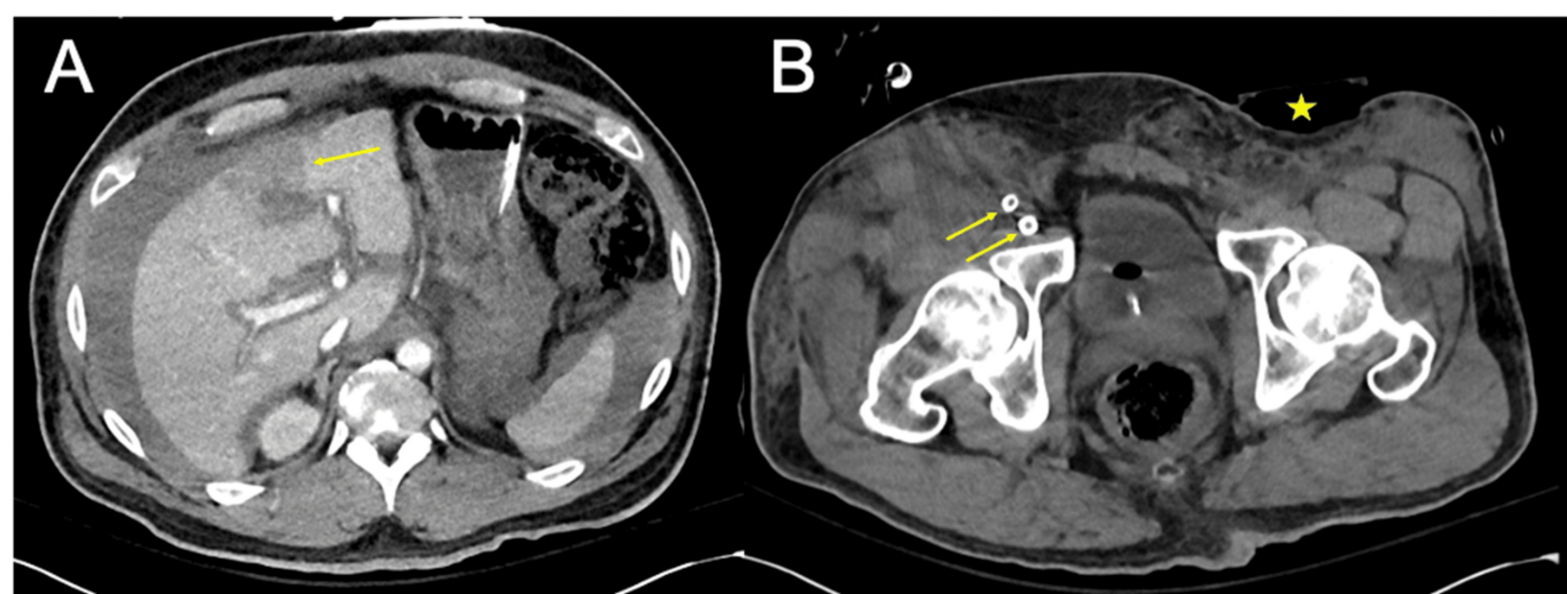
Arterial and delayed phase contrast-enhanced computed tomography (CT). Axial (A) CT scan in the arterial phase demonstrating a liver laceration involving segment IV (yellow arrow), without evidence of active contrast extravasation, and (B) delayed-phase contrast-enhanced CT demonstrating right common femoral artery and vein cannulation for ECMO support (yellow arrows), with a femoral compression (FemoStop; Temecula, CA: Abbott) device (yellow star) over the left groin. ECMO: extracorporeal membrane oxygenation

Computed tomography (CT) of the abdomen and pelvis demonstrated a small segment IV liver laceration, likely secondary to cardiopulmonary resuscitation, with a moderate hemoperitoneum of approximately 500 mL, without evidence of active contrast extravasation (Figure 1A). CT imaging also showed the right common femoral artery and vein ECMO cannulas and the FemoStop compression device overlying the left groin (Figure 1B). At the time of imaging, the patient had already received 6 units of packed red blood cells and had a hemoglobin of 8.5 g/dL. Following CT imaging, given the concern for ongoing hemorrhage and the need for systemic anticoagulation for VA-ECMO, interventional radiology (IR) was consulted for angiographic evaluation of the liver and left iliofemoral system.

Diagnostic evaluation

Given that the right common femoral vessels were occupied by VA-ECMO cannulas and the FemoStop compression device remained in-situ over the left groin, bilateral femoral access was limited. Because the liver laceration identified on CT represented the primary suspected source of hemorrhage, arterial access was obtained via the left radial artery. Celiac angiography demonstrated no evidence of active hepatic arterial extravasation.

**Figure 2:**
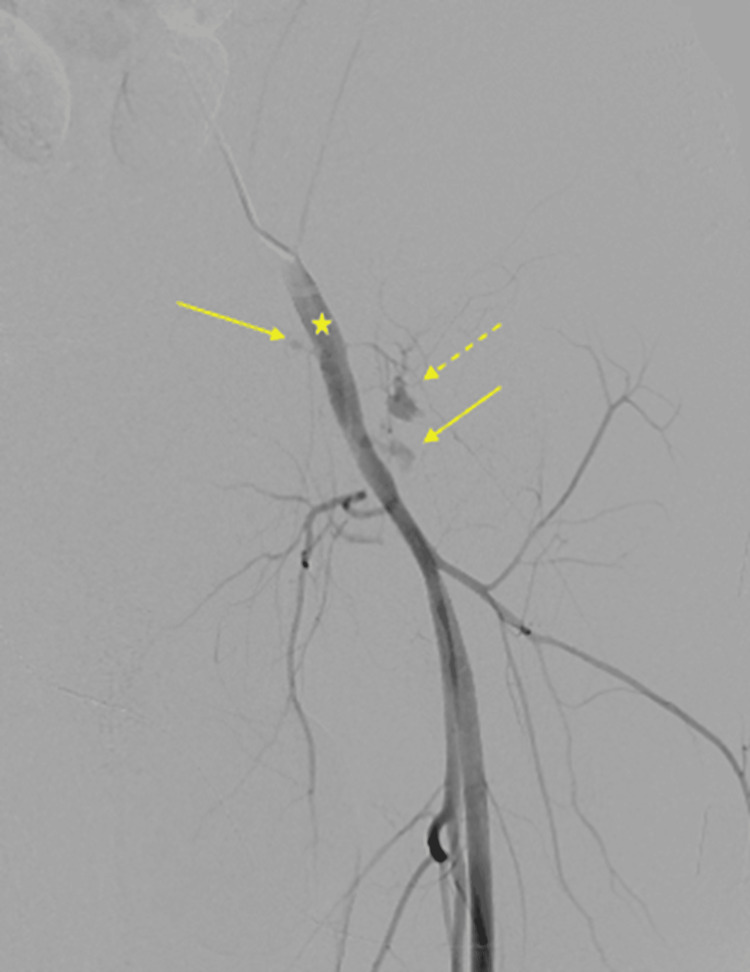
Angiogram of the left external iliac artery demonstrating multiple sites of active extravasation (solid arrows) in the common femoral artery (star) and small muscular branch (dash arrow).

Due to the concern for ongoing hemorrhage, despite a negative visceral angiogram, and the known history of traumatic left groin access attempts, dedicated angiography of the left iliofemoral system was performed. This revealed multiple sites of active contrast extravasation from the left common femoral artery and a small muscular branch, consistent with iatrogenic arterial injury (Figure 2).

Intervention

**Figure 3:**
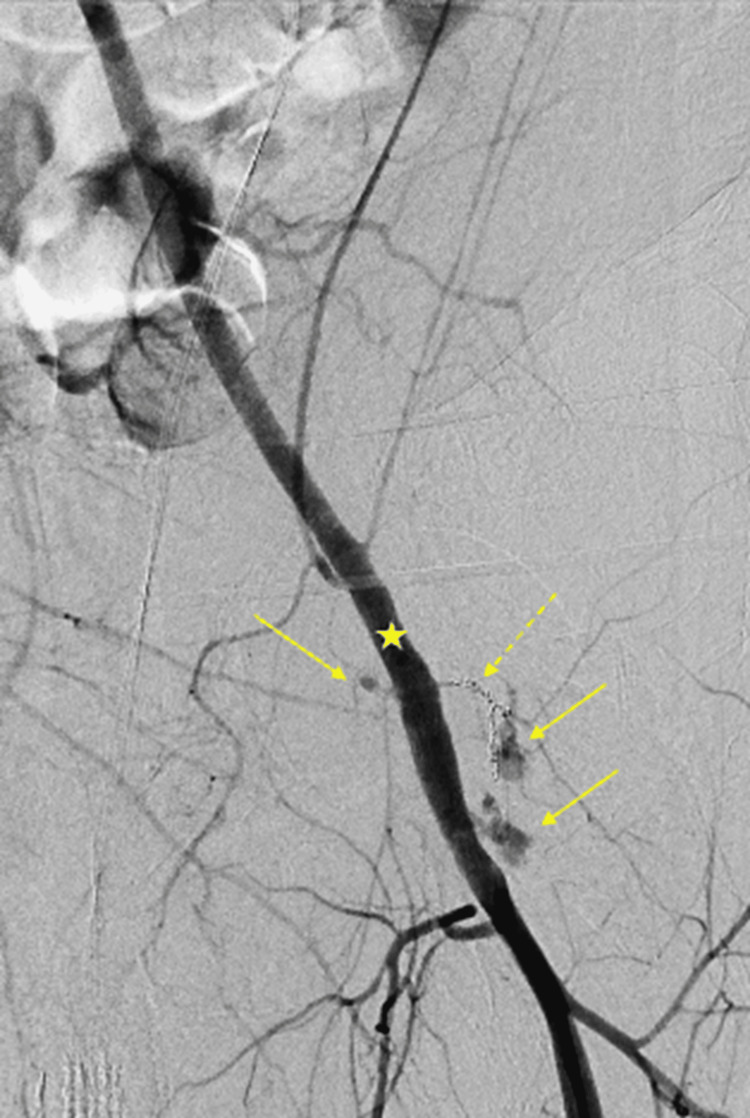
Angiogram from the left external iliac artery after coil embolization (dash arrow) showing persistent contrast extravasation (solid arrow) from the common femoral artery (star).

Initially, a selective embolization was performed of the small muscular branch arising from the CFA using two 2x40 mm Embold detachable coils (Marlborough, MA: Boston Scientific) (Figure 3). Despite the embolization, angiography demonstrated persistent multifocal extravasation from the left CFA and muscular branches.

Treatment options were limited, and additional femoral access attempts and prolonged compression risked worsening the hemorrhage. Surgical repair was considered prohibitively high risk due to VA-ECMO support and coagulopathy. Contralateral crossover was not feasible due to ECMO cannulation in the right groin, and direct left femoral access was precluded by the in-situ FemoStop device. Although the left radial approach had permitted diagnostic angiography, the required covered stent exceeded the working length achievable from radial access, precluding graft delivery via this route.

Given these constraints, a salvage retrograde pedal access strategy was pursued. Posterior tibial access was selected due to favorable vessel caliber and improved ultrasound visualization in the setting of diffuse anasarca. Ultrasound-guided access of the left posterior tibial artery was obtained using a 21-gauge micropuncture needle. Access was obtained using the Seldinger technique, and a 6-French Glidesheath Slender (Tokyo, Japan: Terumo Corp.) was placed.

**Figure 4:**
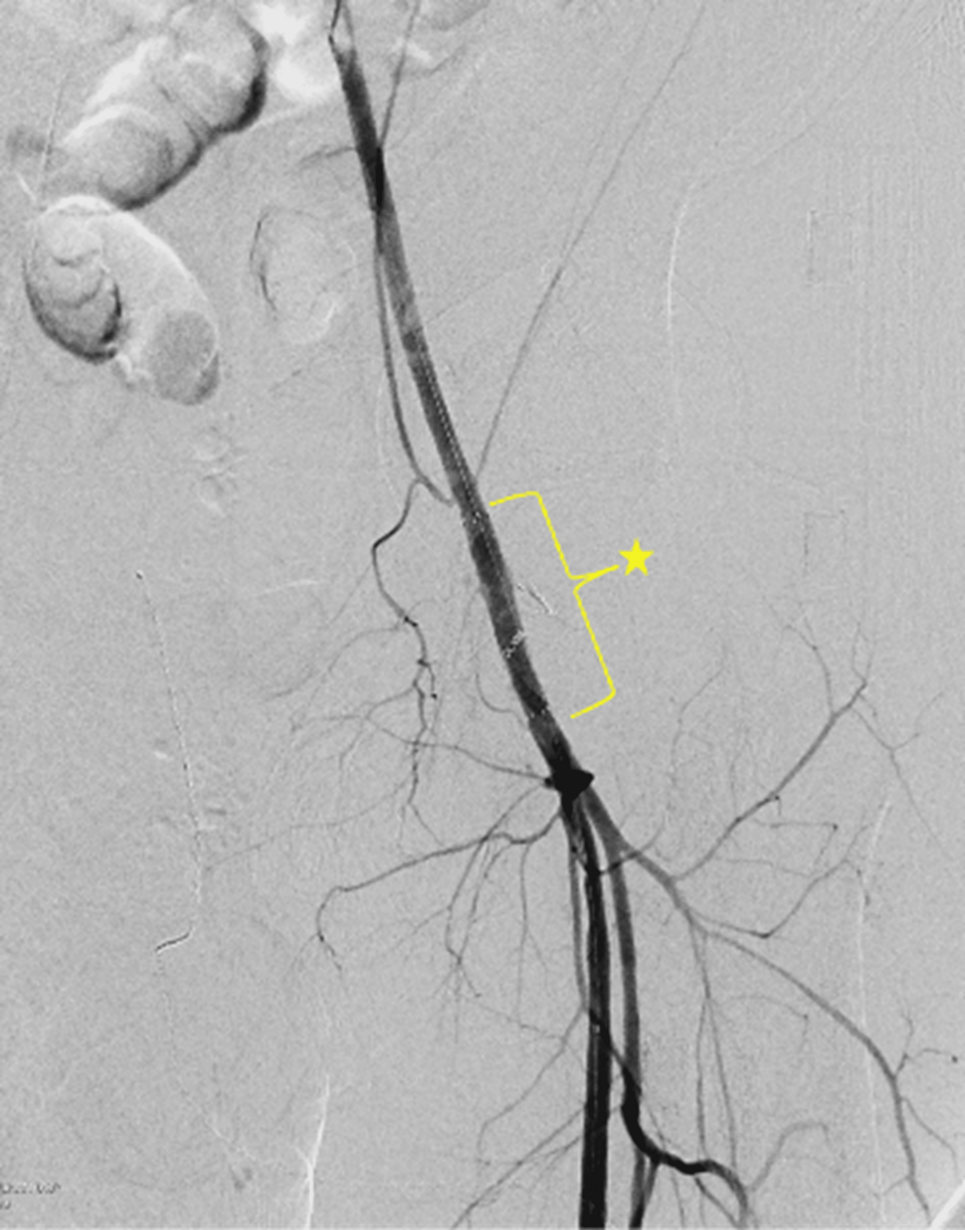
Angiogram after deployment of a 6×50 mm covered stent across the injured common femoral artery (star). Post-treatment angiogram of the left external iliac artery showing deployment of the 6x50 mm covered Viabahn stent (Flagstaff, AZ: W. L. Gore & Associates) across the injured common femoral artery (star) with resolution of contrast extravasation sites and preserved blood flow to the left lower limb.

A 5-Fr Berenstein (Bern) catheter (South Jordan, UT: Merit Medical Systems, Inc.) and a 0.035-inch Bentson guidewire (Bloomington, IN: Cook Medical) were advanced retrograde across the left common femoral artery into the abdominal aorta. After angiographic reassessment and vessel sizing, a 6x50 mm covered self-expandable Gore Viabahn endoprosthesis stent (Flagstaff, AZ: W. L. Gore & Associates) was advanced from the posterior tibial access and successfully deployed across the injured segment of the left common femoral artery. Completion angiography demonstrated complete resolution of contrast extravasation with preserved flow to the left lower extremity (Figure 4).

Outcome

The patient tolerated the procedure without immediate complications. Hemostasis at the posterior tibial access site was achieved with manual compression. The left radial access was maintained for a subsequent cardiology procedure. Systemic anticoagulation with heparin was initiated once the bleeding stopped and maintained during VA-ECMO support. During the nine-day hospital course, the patient had no recurrent bleeding, limb ischemia, or need for reintervention. Subsequent surgical exploration demonstrated hepatic injury without active bleeding, confirming the common femoral artery injury as the primary source of hemorrhage. A detailed timeline of the patient's clinical course, including the hepatic injury and subsequent resuscitative efforts, is summarized in Table 1.

**Table 1:** Timeline of the patient's clinical course. IR: interventional radiology; VA-ECMO: venoarterial extracorporeal membrane oxygenation; STEMI: ST-elevation myocardial infarction; CFA: common femoral artery

Clinical timeline			
Step	Event	Clinical details	Iatrogenic consequence
Cardiac arrest	Witnessed collapse with acute dyspnea	Inferoseptal STEMI; ventricular fibrillation cardiac arrest en route to hospital	-
Resuscitation: cardiac massage	Advanced cardiac life support (ACLS) with multiple defibrillations; return of spontaneous circulation (ROSC) achieved	Prolonged CPR with chest compressions required	Segment IV liver laceration with moderate hemoperitoneum (CPR-related)
Resuscitation: femoral access attempts	Multiple unsuccessful attempts at left groin arterial access by the emergency/rescue team	Femoral compression device (FemoStop Gold; Temecula, CA: Abbott) applied for hemostasis after failed attempts	Multifocal iatrogenic injury to the left common femoral artery (left CFA) and a small muscular branch
VA-ECMO placement	Venoarterial ECMO cannulation for refractory cardiogenic shock	Right common femoral artery and vein cannulated; systemic anticoagulation anticipated	Precluded contralateral crossover approach and surgical repair (high operative risk in settings of ECMO and coagulopathy)
Diagnostic angiography: left radial approach	IR consultation; left radial artery access obtained (bilateral femoral access unavailable)	Celiac angiography: no active hepatic arterial extravasation	Confirmed primary hemorrhage source was the left CFA; multiple sites of active contrast extravasation from left CFA and muscular branch identified
Coil embolization	Selective embolization of the small muscular branch from the left CFA	Two 2×40 mm Embold detachable coils (Marlborough, MA: Boston Scientific) deployed	Persistent multifocal extravasation from the left CFA remained despite embolization
Retrograde pedal access and covered stent	Ultrasound-guided left posterior tibial artery access (21G micropuncture; 6-Fr Glidesheath Slender, Tokyo, Japan: Terumo Corp.); retrograde approach to left CFA	5-Fr Berenstein catheter (South Jordan, UT: Merit Medical Systems, Inc.) + 0.035-inch Bentson wire (Bloomington, IN: Cook Medical) advanced retrograde into abdominal aorta; 6×50 mm Gore Viabahn (Flagstaff, AZ: W. L. Gore & Associates) covered stent deployed across injured left CFA segment	Complete resolution of contrast extravasation; preserved left lower extremity perfusion on completion angiography

## Discussion

Iatrogenic injury to the CFA is a recognized complication of percutaneous vascular access and can commonly occur when arterial access is obtained under suboptimal conditions [3-5]. In critically ill patients, surgical repair may be limited by coagulopathy and hemodynamic instability [2,10]. Furthermore, external compression techniques may exacerbate or be insufficient to control the bleeding [1,10]. Consequently, the use of minimally invasive endovascular alternatives, including covered stent-grafts and embolization techniques, has increased [1,5,11].

Endovascular repair with covered stent-grafts has emerged as a minimally invasive alternative for critically ill or hemodynamically unstable patients who are unable to tolerate open surgery and to mitigate the risks associated with surgical repair [1,10]. This technique can be performed under local anesthesia and moderate sedation, further avoiding the morbidity associated with open surgical procedures [1,4,5]. Recent single-center studies have reported technical success rates of 95-97.5% for covered stent repair of iatrogenic femoral artery injuries, with 100% stent graft patency at 30 days and one year in surviving patients [4,5]. Furthermore, a pooled literature review of 585 patients across 15 studies confirmed near-100% technical success with preserved patency at follow-up ranging from three months to three years [4]. Although antegrade or contralateral femoral access is the commonly used route for delivering a stent graft, these pathways may be unavailable when large-bore devices such as ECMO cannula or other indwelling catheters occupy the access site [1]. In such cases, retrograde pedal or tibial access has been reported as a bailout route for hemorrhage control when conventional femoral entry is not an option [12-14]. This approach aligns with the recent literature favoring endovascular techniques for urgent hemorrhage control in hemodynamically unstable patients [4,15].

In our patient, conventional femoral access was not feasible due to the presence of a right groin VA-ECMO cannula and a compression device on the left groin. Furthermore, although radial access was adequate for diagnostic angiography, it could not accommodate the delivery of a covered stent because commercially available devices lack sufficient shaft length to reach the CFA. It is important to note that when covered stent delivery via the transradial route is not feasible, balloon occlusion via radial crossover can be performed as a bailout strategy to achieve temporary hemostasis at the femoral access site, while a new femoral access is simultaneously established for definitive covered stent repair [9]. However, this approach requires the availability of at least one femoral access site for stent delivery. In our case, femoral access was not available, and radial balloon occlusion would have provided only temporary hemostasis without a route for definitive repair. Therefore, a retrograde posterior tibial access approach was selected, enabling navigation to the common femoral artery and successful deployment of a covered stent. The outcome of this case is consistent with the high technical success and low complication rates reported in larger series of endovascular femoral artery repair [4,5]. This case underscores the importance of systematic evaluation of all available access routes, the utility of distal retrograde access as a bailout strategy, and the value of multidisciplinary decision-making. However, the generalizability of these findings is limited, as this represents a single case from a single institution. Finally, it is important to acknowledge that the endovascular approach described in this report depends on the availability of specialized equipment, trained personnel, and multidisciplinary critical care infrastructure. In resource-limited settings, including low- and middle-income countries (LMICs) where covered stent-grafts, fluoroscopy, and hybrid operative capabilities may not be accessible, open surgical repair may represent the only available option for definitive hemorrhage control [16,17].

## Conclusions

This case illustrates how retrograde pedal access can serve as a viable salvage strategy for iatrogenic CFA hemorrhage when conventional access routes are unavailable. In these settings, effective treatment depends on procedural planning, understanding device limitations, and familiarity with alternative access techniques. However, further reports and dedicated multi-institutional research are required to validate the generalizability of this technique.
